# ViNe-Seg: deep-learning-assisted segmentation of visible neurons and subsequent analysis embedded in a graphical user interface

**DOI:** 10.1093/bioinformatics/btae177

**Published:** 2024-04-03

**Authors:** Nicolas Ruffini, Saleh Altahini, Stephan Weißbach, Nico Weber, Jonas Milkovits, Anna Wierczeiko, Hendrik Backhaus, Albrecht Stroh

**Affiliations:** Institute for Human Genetics, University Medical Center, Johannes Gutenberg University, 55131 Mainz, Germany; Leibniz Institute for Resilience Research, Leibniz Association, 55122 Mainz, Germany; Leibniz Institute for Resilience Research, Leibniz Association, 55122 Mainz, Germany; Institute of Developmental Biology and Neurobiology (iDN), Johannes Gutenberg University, 55128 Mainz, Germany; Fraunhofer Institute for Industrial Mathematics ITWM, 67663 Kaiserslautern, Germany; Institute of Developmental Biology and Neurobiology (iDN), Johannes Gutenberg University, 55128 Mainz, Germany; Institute for Human Genetics, University Medical Center, Johannes Gutenberg University, 55131 Mainz, Germany; Leibniz Institute for Resilience Research, Leibniz Association, 55122 Mainz, Germany; Leibniz Institute for Resilience Research, Leibniz Association, 55122 Mainz, Germany; Leibniz Institute for Resilience Research, Leibniz Association, 55122 Mainz, Germany

## Abstract

**Summary:**

Segmentation of neural somata is a crucial and usually the most time-consuming step in the analysis of optical functional imaging of neuronal microcircuits. In recent years, multiple auto-segmentation tools have been developed to improve the speed and consistency of the segmentation process, mostly, using deep learning approaches. Current segmentation tools, while advanced, still encounter challenges in producing accurate segmentation results, especially in datasets with a low signal-to-noise ratio. This has led to a reliance on manual segmentation techniques. However, manual methods, while customized to specific laboratory protocols, can introduce variability due to individual differences in interpretation, potentially affecting dataset consistency across studies. In response to this challenge, we present ViNe-Seg: a deep-learning-based semi-automatic segmentation tool that offers (i) detection of visible neurons, irrespective of their activity status; (ii) the ability to perform segmentation during an ongoing experiment; (iii) a user-friendly graphical interface that facilitates expert supervision, ensuring precise identification of Regions of Interest; (iv) an array of segmentation models with the option of training custom models and sharing them with the community; and (v) seamless integration of subsequent analysis steps.

**Availability and implementation:**

ViNe-Seg code and documentation are publicly available at https://github.com/NiRuff/ViNe-Seg and can be installed from https://pypi.org/project/ViNeSeg/.

## 1 Introduction

Functional two-photon calcium imaging allows for a single-cell resolution readout of neuronal circuit function comprising several hundred neurons, e.g. in the rodent cortex. This functional microcircuit imaging technique is capable of revealing subtle and early circuit dysfunction in mouse models of neurological diseases (Grienberger and Konnerth 2012, Ellwardt *et al.* 2018). To identify the activity signature of a microcircuit, information about phases of activity and periods of quiescence of all neurons in the field of view is necessary. Here, the identification and binarization of putative action potential-related calcium transients as a correlate of suprathreshold neuronal activity is the basis for the application of analytical methods originally established in electrophysiology (Fu *et al.* 2021). Therefore, the segmentation of neuronal somata, i.e. the separation of the cells’ signal from the image background, into regions of interest (ROIs), is an essential prerequisite for subsequent analysis.

**Figure 1.**
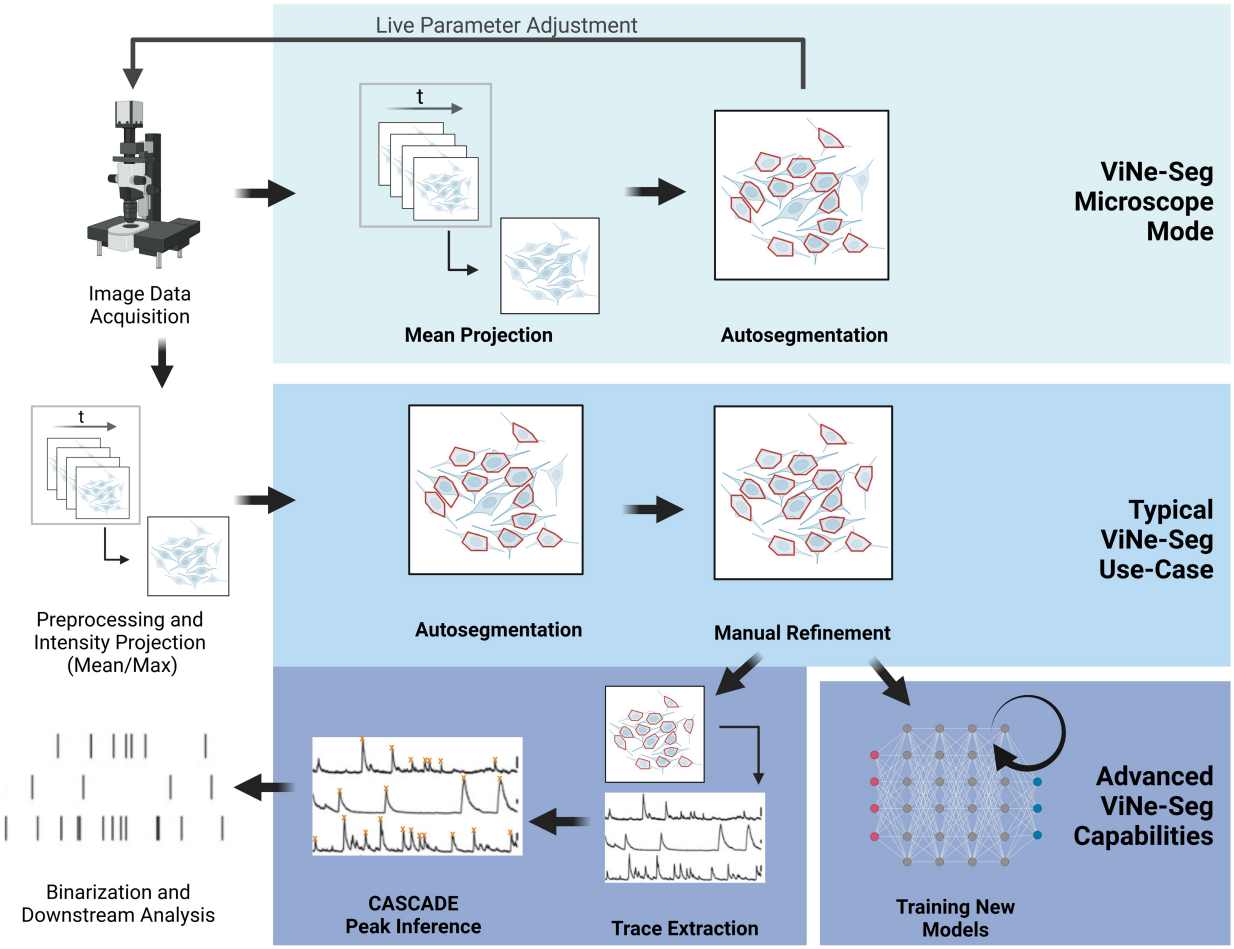
Integration of ViNe-Seg into typical Calcium Imaging Analysis workflows. ViNe-Seg offers a broad spectrum of analytical functionalities. At its heart, it is designed for semi-automatic neuronal segmentation, typically relying on the mean or max projections of fully executed experiments. For those well-versed in machine learning, ViNe-Seg provides the flexibility to use the semi-automatically labeled images for further model training, with the necessary scripts made available on GitHub. Beyond segmentation, the platform is equipped to handle tasks like trace extraction, df/f estimation, and CASCADE Peak Inference (Rupprecht *et al.* 2021), especially when users engage the advanced mode of ViNe-Seg. Furthermore, there is an integrated ‘Microscope Mode’ which is invaluable for researchers aiming to make real-time adjustments to experimental parameters. In this mode, ViNe-Seg actively calculates the mean projection of a running experiment and oversees the segmentation procedure, granting users a dynamic feedback loop to refine their methodologies as the experiment progresses. Figure created using BioRender.

Here, we propose a user-integrating deep learning-based approach, ViNe-Seg, for semi-automatic segmentation of functional two-photon calcium imaging datasets with two key features: Firstly, ViNe-Seg identifies both, active and inactive neuronal somata (Fig. 1), built upon a graphical user interface (GUI), and, secondly, ViNe-Seg allows the expert user to refine and correct the segmentation output. In addition, subsequent analysis steps are integrated such as trace extraction, ΔF/F estimation, and peak detection.

## 2 Implementation

ViNe-Seg is written in Python, makes use of YOLOv8 (Jocher *et al.* 2022), a deep-learning model to automatically segment images, and is embedded in a GUI that is derived from the manual segmentation software labelme (https://github.com/wkentaro/labelme). The basic version of ViNe-Seg can be easily installed using the command pip install vineseg (https://pypi.org/project/ViNeSeg/). ViNe-Seg is trained for aggregated images from experiments where commonly used methods include max or mean projections. Along with our segmentation code, we provide functionalities for spike inference and trace extraction by integrating the CASCADE tool (Rupprecht *et al.* 2021) and code from the Allen Software Development Kit (SDK) (https://github.com/AllenInstitute/AllenSDK), respectively.

*Training of our models* was performed using a dataset of labeled mean and max images from three sources: 1329 publicly available labeled images using the Allen SDK Brain Observatory Cache (de Vries *et al.* 2020, Siegle *et al.* 2021) as well as 19 labeled images of the Neurofinder dataset (https://github.com/codeneuro/neurofinder), and 449 images simulated with the neural anatomy and optical microscopy (NAOMi) tool (Song *et al.* 2021). We split the datasets into 85% training images, 5% validation images, and 10% test images. The segmentation is performed with the small model of YOLOv8. We chose YOLOv8 for ViNe-Seg due to its fast inference speed, low resource demands, and reliable prediction accuracy. We trained the YOLOv8 small default model for 50 epochs on each respective dataset and one model on all datasets at the same time. The robustness is increased during the training by applying several data augmentation techniques. We selected the best-performing model based on the validation set and assessed the performance on the test set. On the most extensive test dataset, the Allen SDK Brain Observatory Cache dataset, we achieved an F1 score of 0.84 and a mAP50 of 0.88 (all F1 scores for the respective trained models are shown in Supplementary Table S1). The performance of the model depends on the similarity between the training data and the test data. In two-photon microscopy, the images can vary significantly between labs, therefore it is advantageous to train a custom model for each lab’s microscope images. We provide an easy-to-use training script along with a detailed explanation to train a model on a custom dataset, which can also be found on the ViNe-Seg GitHub page (https://github.com/NiRuff/ViNe-Seg).

## 3 Usage

The trained models can be started within the GUI when an aggregated image (TIF or PNG format) is loaded. The software outputs the segmentation results as a JSON file, which is automatically loaded into the GUI, displaying the polygonal positions of all ROIs.

Users have the flexibility to modify the segmentation process in multiple ways. They can select an appropriate threshold for the model’s confidence score in identifying neuronal somata or make manual adjustments by adding or editing polygonal cell selections. This makes the segmentation process inherently semi-automatic. The refined labeled dataset not only serves as the basis for further post-analytical procedures but holds the potential for enhancing the used model for subsequent datasets. For explicit training methodologies, we direct readers to our comprehensive documentation on GitHub. Researchers who have developed own segmentation models, especially using comprehensive databases, are encouraged to contact us to integrate helpful models into the ViNe-Seg model manager, ensuring broader accessibility to the ViNe-Seg academic community.

Additionally, ViNe-Seg offers the trace extraction and baseline correction of the chosen ROIs, making each ROIs ΔF/F values available. The subsequent peak detection is implemented with CASCADE (Rupprecht *et al.* 2021) but ViNe-Seg provides the flexibility to easily integrate a custom algorithm for this step. Furthermore, we integrated a microscope mode in ViNe-Seg, in which the user, during an ongoing experiment, can generate an average intensity projection of the latest up to 500 acquired images and perform the segmentation on this image. Thus, preliminary information about the acquired data can be collected during the experiment to adjust experimental parameters during data acquisition if necessary.

## 4 Discussion

### 4.1 Variability in image segmentation

Computational support in image segmentation holds the potential to significantly enhance consistency and accelerate analyses. Human experts, as shown in (Giovannucci *et al.* 2019), can exhibit notable inter-observer variability, sometimes resulting in precision values as low as 0.77, highlighting the need for more automation in segmentation.

### 4.2 Trade-offs between fully- and semi-automatic approaches

However, fully automated approaches, such as STNeuroNet (Giovannucci *et al.* 2019), CaImAn (Vogt 2019), and the more recent NeuroSeg-II (Xu *et al.* 2023) that primarily focus on active neuron labeling using Ca2+ trajectories, are not immune to the identification of false positive neuronal somata. At the same time, semi-automatic methods, like those used in computer tomography data segmentation (Dinkel *et al.* 2013), have been shown to reduce the inter-observer variability effectively, despite involving human experts in the segmentation process. In this context, expert user interaction in semi-automated processes can at the same time improve performance and minimize such errors.

### 4.3 The Vine-Seg approach

1. ViNe-Seg aims to segment all visible neuronal somata, not just the active ones, providing a comprehensive view that other auto-segmentation tools might miss. This is pivotal in experiments where tracking the same neuronal instances across different conditions or time points is required, regardless of their activation state. While in principal also models trained for active neuron segmentation can be run in ViNe-Seg, the initial models and the design of ViNe-Seg are shaped for visible neuron segmentation.
2. Contrasting with typical post hoc analysis tools, ViNe-Seg’s unique Microscope Mode can be activated right at the experiment’s onset. This early integration allows for real-time adjustments, a feature not commonly found in similar segmentation tools. Although ViNe-Seg does not offer online segmentation capabilities like CITE-ON (Sità *et al.* 2022), it considerably narrows the gap between experiment and subsequent analysis, enabling near real-time imaging and interventions within a semi-automatic framework.
3. ViNe-Seg distinguishes itself by integrating user participation directly into the segmentation workflow, addressing the limitations observed in fully automated approaches. This strategy crucially lowers the risk of endorsing erroneous segmentations, thereby elevating the confidence researchers place in their data analysis. Furthermore, it opens avenues for the continuous improvement of segmentation models through the integration of manually adjusted data. This iterative refinement process is particularly valuable in the face of diverse and inconsistent data qualities, as evidenced by the cautious predictions with new datasets depicted in Supplementary Fig. S3. These challenges necessitate possible adjustments in model confidence thresholds and underscore the importance of the capability for users to train personalized models. By utilizing data refined within the ViNe-Seg framework, scientists are equipped to closely align segmentation models with their specific experimental demands, especially when faced with novel datasets that test the limits of existing models’ adaptability. This enhancement of model personalization and adaptability through active user involvement positions ViNe-Seg as a versatile and responsive tool in the dynamic field of neuroscience research.

### 4.4 Final notes on user engagement in ViNe-Seg

Distinctively positioned between manual and automated methods, ViNe-Seg integrates user-friendly manual adjustments into its workflow. This user-friendly design empowers active engagement in segmentation, allowing for intuitive modifications and model selection. Such involvement ensures a balance between the speed of automation and the precision of expert input, enhancing the interaction and control of the experimenter and leading to more accurate, supervised results.

## Supplementary Material

btae177_Supplementary_Data

## Data Availability

The data underlying this article are available in the article, its online supplementary material and in https://github.com/NiRuff/ViNe-Seg.
