# Supplementary material for: ViNe-Seg: deep-learning-assisted segmentation of visible neurons and subsequent analysis embedded in a graphical user interface
Source: Bioinformatics. 2024 Apr 3;40(4):btae177. doi: 10.1093/bioinformatics/btae177 (PMC11034984; doi:10.1093/bioinformatics/btae177)
Supplement: btae177_Supplementary_Data [file btae177_supplementary_data.docx]

**Supplementary Table 1**

Performance of different models on various test datasets. ViNe-Seg models were trained on Allen Brain Observatory Data (see Reference 1-5), NAOMi Simulated Data (see Reference 6), and Neurofinder Data (https://github.com/codeneuro/neurofinder) or subsets of those as described in the Training Dataset column. For all ViNe-Seg models datasets were split in 85% training images, 5% validation images, and 10% test images. Shown metrices correspond to segmentation results when applying a 0.5 confidence threshold.

| **Model** | **Training Dataset** | **Test Dataset** | | | |
| --- | --- | --- | --- | --- | --- |
|  |  | **Allen** | | **Simulated** | |
|  |  | mAP50 | F1 | mAP50 | F1 |
| YOLOv8 small | Allen, simulated, neurofinder | Box: 0.85845  Seg: 0.86852 | Box: 0.77007  Seg: 0.80792 | Box: 0.74291  Seg: 0.73167 | Box: 0.74297  Seg: 0.72930 |
| YOLOv8 small | Allen | Box: 0.90081  Seg: 0.87844 | Box: 0.84030  Seg: 0.83617 | Box: 0.12690  Seg: 0.20469 | Box: 0.18242  Seg: 0.25722 |
| YOLOv8 small | Simulated | Box: 0.27357  Seg: 0.32124 | Box: 0.25074  Seg: 0.29868 | Box: 0.75679  Seg: 0.74087 | Box: 0.75991  Seg: 0.74463 |


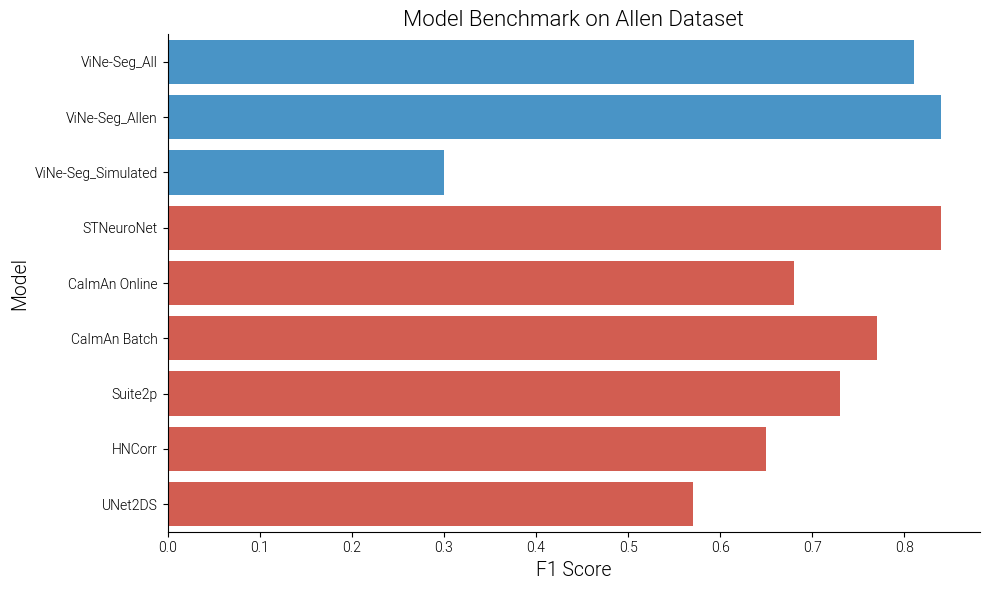


**Supplementary Figure 1**

Performance of different models on various test datasets. ViNe-Seg models were trained on Allen Brain Observatory Data(see Reference 1-5), NAOMi Simulated Data (see Reference 6), and Neurofinder Data (https://github.com/codeneuro/neurofinder) as described in Table S1. F1 values for other models than the ViNe-Seg models are derived from the STNeuroNet paper’s benchmarking (see Reference 8, Table S2).


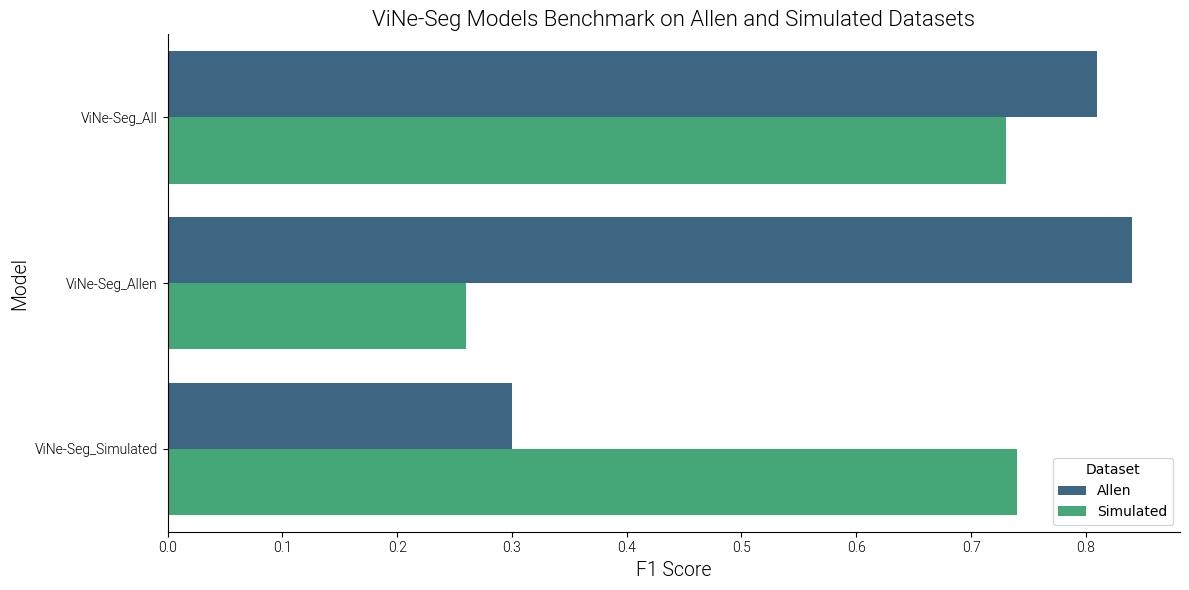


**Supplementary Figure 2**

Performance of different ViNe-Seg models on Allen Brain Data and Simulated Data. The models trained specifically on one data origin perform best when being applied to new unseen data of the same source. However, the ViNe-Seg_All model generalizes well to test sets of all data origins.


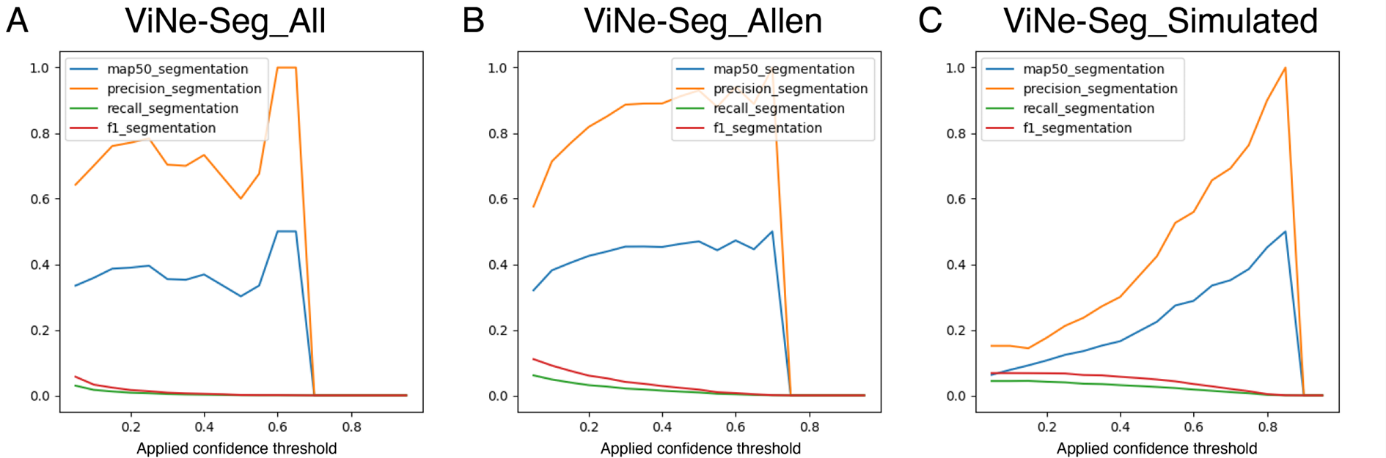


**Supplementary Figure 3**

Performance of different models on Neurofinder test datasets applying various confidence thresholds. ViNe-Seg models were trained as described in Supplementary Table 1. The predictions of the three included ViNe-Seg models show a general tendency towards conservative predictions exemplified by low recall and high precision values. Neurofinder data is based on data originating from four laboratories, varying imaging conditions, cortical and subcortical regions and a mix of visible and active neuron labeling.

**References**

Allen Brain Data

1. de Vries, Saskia EJ, et al. "A large-scale standardized physiological survey reveals functional organization of the mouse visual cortex." Nature neuroscience 23.1 (2020): 138-151.
2. Neuropixels Visual Coding Available online: https://knowledge.brain-map.org/data/4YYLRZZGK82FQ85NIH8/summary (accessed on 28 March 2022).
3. Visual Coding Ophys Available online: https://knowledge.brain-map.org/data/ILO10QI5VTDFVWYBQXX/summary (accessed on 28 March 2022).
4. Visual Behavior Available online: https://knowledge.brain-map.org/data/ZQ5EP552UXR0LI83B66/summary (accessed on 28 March 2022).
5. Siegle, Joshua H., et al. "Survey of spiking in the mouse visual system reveals functional hierarchy." Nature 592.7852 (2021): 86-92.

NAOMi Simulated Data

1. Song, Alexander, et al. "Neural anatomy and optical microscopy (NAOMi) simulation for evaluating calcium imaging methods." Journal of neuroscience methods 358 (2021): 109173.

Neurofinder Data

1. http://neurofinder.codeneuro.org /

Benchmarked algorithms

1. Soltanian-Zadeh, S., Sahingur, K., Blau, S., Gong, Y., & Farsiu, S. (2019). Fast and robust active neuron segmentation in two-photon calcium imaging using spatiotemporal deep learning. Proceedings of the National Academy of Sciences of the United States of America, 116(17), 8554–8563.
2. Giovannucci, A., Friedrich, J., Gunn, P., Kalfon, J., Brown, B. L., Koay, S. A., Taxidis, J., Najafi, F., Gauthier, J. L., Zhou, P., Khakh, B. S., Tank, D. W., Chklovskii, D. B., & Pnevmatikakis, E. A. (2019). CaImAn an open source tool for scalable calcium imaging data analysis. eLife, 8, e38173. https://doi.org/10.7554/eLife.38173
3. Marius Pachitariu, Carsen Stringer, Mario Dipoppa, Sylvia Schröder, L. Federico Rossi, Henry Dalgleish, Matteo Carandini, Kenneth D. Harris, (2017), Suite2p: beyond 10,000 neurons with standard two-photon microscopy, bioRxiv 061507; doi: https://doi.org/10.1101/061507
4. Spaen, Q., Asín-Achá, R., Chettih, S. N., Minderer, M., Harvey, C., & Hochbaum, D. S. (2019). HNCcorr: A Novel Combinatorial Approach for Cell Identification in Calcium-Imaging Movies. eNeuro, 6(2), ENEURO.0304-18.2019. https://doi.org/10.1523/ENEURO.0304-18.2019
